# Employer Attractiveness of EMNEs: The Role of CSR in Overcoming Country-of-Origin Image Constraints in Developed Host Countries

**DOI:** 10.1007/s11575-022-00498-7

**Published:** 2022-12-07

**Authors:** Bich Ngoc Le, Dirk Morschett

**Affiliations:** University of Fribourg, Boulevard de Pérolles 90, Fribourg, Switzerland

**Keywords:** Emerging market multinational enterprises, Job-pursuit intention, International human resource management, Corporate social responsibility, CSR

## Abstract

Attracting a qualified workforce is a challenge for all companies but in particular for foreign subsidiaries of emerging market MNEs (EMNEs) in developed countries due to their double disadvantages of liability of foreignness plus liability of emergingness. Based on signaling theory, this study investigates whether corporate social responsibility (CSR) contributes to overcoming these liabilities. A web-based experiment with realistic recruitment webpages, involving 490 potential applicants from Germany, Switzerland and Austria, reveals that potential applicants in developed host countries have lower job-pursuit intention to EMNEs than to developed market MNEs, but that good CSR helps mitigate this negative effect. Nonetheless, we find that applicants are not intensively searching for CSR information on a recruitment webpage, constituting an impediment in EMNEs’ attempts to reap maximum benefits from their CSR engagement.

## Introduction

Emerging market multinational enterprises (EMNEs) have increasingly expanded their operations worldwide, not only in other emerging markets but also in developed countries (Hennart, 2012; Shirodkar & Shete, 2022). Nevertheless, these EMNEs often lack firm-specific assets like internalized knowledge (Meyer & Xin, 2018). Thus, they need to attract local talent with the right know-how to develop the firm-specific advantages that they have so far lacked in competition with more advanced competitors from Western countries (Held & Bader, 2018; Law et al., 2009).

Acquiring qualified workforce is crucial for all MNEs in the ever-intensifying competition for scarce highly skilled employees, called “war for talent.” Indeed, companies are struggling more than ever to fill open positions – nearly 7 in 10 employers worldwide cannot find the right skills they need, which is the highest value in the last 15 years, according to Manpower Group’s annual Talent Shortage Survey (ManpowerGroup, 2022). This issue is more relevant and challenging for EMNEs since they suffer from double disadvantages of liabilities of foreignness plus liabilities of emergingness (Zhang et al., 2020a). Therefore, the question arises as how EMNEs can compete with developed market multinational enterprises (DMNEs) in the labour markets of developed countries and become more attractive to prospective applicants. So far, researchers have closely looked into applicant attraction of DMNEs. However, little attention is devoted to how EMNEs can attract and acquire local potential employees in developed countries (Held & Bader, 2018; Meyer & Xin, 2018). Addressing this question is vital to the continuing international growth of EMNEs. Based on a report of UNCTAD (2019), outward investment of developing economies was $418 billion, accounting for 41% of the global outflows. The number of EMNEs in Fortune Global 500 also increased more than twofold, from 69 in 2007 to 164 in 2017 (Luo & Tung, 2018). In Forbes Global 2000, China has grown from only 43 companies in 2003 to 351 in 2022 (Forbes, 2022).

To overcome country-of-origin image constraints on hiring local talent in developed markets, EMNEs have been recommended to send out signals about otherwise unobservable characteristics of the firms, such as good human resource (HR) practices (Holtbrügge & Kreppel, 2015) or corporate social responsibility (CSR) (Jones et al., 2014). In particular, previous research has shown that elementary HR practices, like career opportunities or pay, have become less useful to attract talents in developed countries due to the interchangeability of jobs and company profiles within the same industry. Consequently, CSR is becoming more important for firms to differentiate themselves from their competitors and to attract qualified employees (Greening & Turban, 2000; Jones et al., 2014; Zhang et al., 2020b). Therefore, we suggest that CSR is an instrument worth considering for EMNEs to close the attractiveness gap to DMNEs in developed countries. Furthermore, although literature has examined the impact of firms’ CSR on job applicants, drawing upon signaling theory, little is known about the underlying mechanisms and contingency factors. For example, some applicants (the receivers of the signal) may interpret the same CSR signal differently from other receivers stemming from distrust of the firm’s motives. Indeed, Gond et al. (2017) conclude their study by calling for further research that provides “integrative analyses of the drivers of CSR and the boundary conditions and mechanisms underlying individual reactions to CSR.”

Responding to this call, we use an experimental approach and adopt a multi-level perspective to investigate the effect of macro-level country-of-origin, firm-level CSR engagement and micro-level individual skepticism on job-pursuit intention to find out how CSR helps mitigate the negative effect of being an emerging market firm on hiring talent in developed markets. Particularly, we create different hypothetical corporation career webpages based on career pages of real multinational companies and observe applicants’ behaviors on these sites. We chose websites as the communicating means because websites have become an increasingly prevalent source of recruitment information. Prior research, e.g., Behrend et al. (2009); Jones et al. (2014), used website printouts in their studies. Nevertheless, only real, interactive websites allow to track respondents’ behaviors.

This study contributes to the literature in the following ways. First, the study extends the CSR research on employee recruitment by adding a country-level factor, namely country-of-origin. Although companies’ CSR engagement has long been suggested to help attract talent in the literature, previous studies were mainly conducted in the domestic context, particularly in Western countries (Maon et al., 2019), leaving country-level factors an under-researched area. One exception is the study of Hong and Kim (2017), which is set in the international recruiting context. Specifically, they investigated Korean applicants’ job-pursuit intentions to US and Chinese MNE. These findings, however, could be subjected to limited generalizability due to using only two countries of origin, US as developed and China as emerging markets. To fill this gap, we investigate these underlying issues on MNEs from ten countries, five developed and five emerging markets, operating in Switzerland, Germany, and Austria. These three host countries are highly developed German-speaking countries, all in the top 25 most attractive destinations for FDI for 5 years in a row, according to Kearney (2019)’s FDI Confidence Index, representing an appropriate study context.

Second, the study brings the concept of country-of-origin image from marketing into international recruitment research. To date, most conceptualization focuses primarily on the relationship between country images and consumer’s product perception despite the fact that country-of-origin image has a broad effect not only on consumers but also on foreign investors or employees (Buhmann & Ingenhoff, 2015).

Third, we take into account applicants’ skepticism, a key challenge of CSR communication (Du et al., 2010). There are two different forms of skepticism: situational skepticism, “which is a momentary state of distrust of an actor's motivations,” and skepticism as a trait of individuals, “which is an individual’s on-going tendency to be suspicious of other people's motives” (Forehand & Grier, 2003). In this study, we focus on the former and control for the later. This will contribute to the advancement of knowledge of individual-level outcomes of CSR. According to a review of Aguinis and Glavas (2012), while differential outcomes of CSR have long been studied, only 4% of the empirical work examined these outcomes at the individual level.

Fourth, we explore the potential of using behavioral experiments to complement the survey-based methods in international business (IB) research. The extant literature has called for the use of experiments as they are mostly absent from the IB research (Zellmer-Bruhn et al., 2016). Thus, we believe that the methodological innovation proposed in this study will make a contribution to the IB literature.

Last but not least, this study addresses applicants’ willingness to search for CSR information of a firm, which is a determinant of their awareness of the firm’s CSR activities. Researchers find that the general public’s awareness of companies’ CSR activities is typically low, representing a serious impediment in firms’ attempts to maximize benefits from CSR engagement (Bhattacharya et al., 2008; Du et al., 2010). The reason is that the general public rarely proactively seeks information about CSR, even for the issues important to them (Dawkins, 2005). Despite its importance, no prior studies addressed willingness to search for CSR information in international recruitment context. Particularly, in previous studies, the authors manipulate CSR by directly giving out information on the company’s CSR activities. However, in reality it is not that straightforward. Therefore, in our study, potential applicants have to actively look for CSR information on companies’ websites to explore their motivation to look for information, especially on CSR.

The study is organized as follows: in the next section, we introduce the theoretical background leading to our hypotheses. We then describe the used methodology. Finally, we present our findings and theoretical as well as practical implications.

## Theoretical Background and Hypotheses

The study is based on signaling theory and on attribution theory. Signaling theory is typically concerned with the use of signals to address information asymmetries between two parties (Rynes, 1989; Spence, 1973). With regard to recruitment context, prospective applicants usually have limited information on a company in the beginning phase of job-choice decision and consequently tend to use the information on hand as signals to reduce initial information asymmetries between the recruiting firms and themselves (Baum & Kabst, 2013). Thus, being the sender of signals, companies often use signals of their interest to convey positive attributes in an effort to influence applicants’ attraction. However, applicants might also be driven by signals which companies can hardly influence. Country-of-origin characteristics, for instance, are not controlled by companies and may serve as signals to shape an individual’s country image, leading to a rather poor job characteristics of companies from emerging markets (Froese et al., 2010; Held & Bader, 2018). For that reason, EMNEs should send out information about their CSR serving as signals in order to be associated with better corporate characteristics.

It is noteworthy, however, that not all applicants (the signal receivers) will interpret the same CSR signal the same way. Some empirical studies have found undesirable effects of CSR initiatives and communications on job applicants, stemming from individual inherent differences and CSR skepticism (Joo et al., 2016; Maon et al., 2019). Thus, it is necessary to add a more nuanced perspective on the effect of CSR signaling. On this basis, attribution theory, which specifically addresses the processes by which individuals evaluate the motives of others and explains how these perceived motives influence subsequent attitudes and behaviors, provides an appropriate analysis framework. Specifically, people will refrain from making correspondent inferences about other’s positive dispositions and engage in more extensive attributional reasoning whenever they have reason to suspect the ulterior motives (Forehand & Grier, 2003; Yoon et al., 2006). This is relevant since positive CSR information of EMNEs, which may be at odds with the expectations of applicants given the negative presumption of emerging country-of-origin image, would further trigger extensive attributional processing, and consequently influence subsequent attitudes and behaviors of the applicants. Our research hypotheses will be further analyzed in the following.

### Country-of-Origin

Researchers have found that MNEs’ country-of-origin has effects on applicants’ perceptions about the attractiveness of MNEs as employers and thus on their job-pursuit intention through country-of-origin image signaling (Froese et al., 2010; Hong & Kim, 2017). Froese et al. (2010) have identified three signals constituting country-of-origin image particularly in the recruiting setting. The first is perception of general human resource practice or norm of companies from a certain country. Previous cross-national studies show that different aspects of employer image are valued differently across different countries and cultures, except for career-enhancing opportunities and good working environment which have unchanged high levels of importance to applicants in varied countries (Alnıaçık et al., 2014; Baum & Kabst, 2013). Since DMNEs are more likely perceived as offering better career-enhancing opportunities and working environment than EMNEs, they would be more attractive to applicants (Froese et al., 2010; Meyer & Xin, 2018).

The second is perception of in-group favoritism of people from a country. According to sociological research, if people from the home country of a MNE exhibit in-group favoritism, they are inclined to favor those who are similar to themselves, e.g. same nationality (Tajfel, 1974). As a result, employees from other countries will be less likely to get promoted compared to those from home country, thus, the less attracted they will be to MNEs from countries with high in-group favoritism. Researchers have found that higher-status, higher-income and more globalized nations, attributes of developed countries, exhibit a lower degree of in-group favoritism, in line with the notion of inequality aversion (Dorrough & Glockner, 2016; Tanaka & Camerer, 2016). Indeed, surveys conducted by Clark and Hoque (2012) reveal that Chinese and Latin Americans, representing emerging nations, show a greater national in-group favoritism than US Americans, epitomizing developed ones. Similarly, in a 73-nation study, Van de Vliert (2011) finds that in-group favoritism is significantly higher in South America, Asia, Africa than in Europe, Canada, the United States, Australia and New Zealand. Therefore, applicants would be less attracted and have less intention to apply to EMNEs because emerging countries are expected to exhibit a higher in-group favoritism.

The final is perception of technological development or industry expertise of a country. Applicants would have a more positive attitude towards technically advanced countries because when working for companies from more advanced countries they can learn from the superior system and upgrade their technological skills. Although nowadays many emerging countries stand out with high growth rates, they still tend to be stereotyped as late developers with low-cost labor, poor transparency and weaker technological and innovative capacities as compared to developed countries (Held & Bader, 2018). This poor country image negatively affects even the world-leading EMNEs, for example the case of Huawei, the Chinese telecoms giant. Despite being a global high-tech leader, it faces doubts and strong opposition in the US and Europe (Zhang et al., 2019). EMNEs, being based in less developed economies, are perceived as less technically advanced as DMNEs, thus, they would be less attractive to applicants.

Derived from the above, country-of-origin image signaling is more likely to be less favorable for EMNEs as compared to DMNEs, leading to a liability of emergingness (Held & Bader, 2018). Thus, applicants would have lower job-pursuit intention to EMNEs. This gives rise to the following hypothesis:

*Hypothesis 1*: Emerging country-of-origin (vs. developed country-of-origin) of an MNE reduces the job-pursuit intention of potential applicants.

### CSR

CSR, which can be broadly defined as “a company’s commitment to minimizing or eliminating any harmful effects and maximizing its long-run beneficial impact on society” (Mohr et al., 2001), is at the forefront on the global corporate agenda in today’s socially conscious market environment (Du et al., 2007). There is evidence from both academic research and marketplace polls that important stakeholders like consumers, employees, and investors incline to take actions to reward good firms and punish bad ones. For instance, Montgomery and Ramus (2011) conducted a study on what MBAs from North America and Europe in the twenty-first century care about during their job search and they found that CSR was ranked in the top five most important factors, being almost as important as salary (relative importance of CSR compared to the top-criterion salary was 95%). In addition, a recent survey of Deloitte (2019) reported that 38% of millennials and Gen Z have backed away from companies that negatively impact the environment and society. Especially, the recent pandemic Covid-19 has brought about an even stronger sense of individual responsibility in both generations with nearly three-fourths intending to take actions to make positive impact on their communities (Deloitte, 2020). They expect business to reflect the same commitment to focusing on social and environmental sustainability. With increasing stakeholders’ expectations and intensifying global competition, firms in general and MNEs in particular are constantly striving to be, or at least to appear, socially responsible (Crifo et al., 2016; Vaara & Tienari, 2008). KPMG (2017) indicates that the G250 CSR reporting rate rocketed from only 35% in 1999 to over 90% in 2017. The question for managers is no longer whether to engage in CSR activities or not, but how to do so (Carroll et al., 2012).

Before analyzing the impacts of CSR on business, it is important to take into account stakeholders’ CSR awareness. Firms cannot yield any good returns to CSR if stakeholders are unaware of their CSR (Du et al., 2010). Stakeholders’ CSR awareness is contingent upon their willingness to search for CSR information. In the job-choice process, applicants will be motivated to process more information about a firm when the benefits from engaging in the information processing outweigh the costs associated with such processing (Cable & Turban, 2001). Thus, they will be more motivated to look for information about a potential employer when they possess little knowledge of the firm than when they have extensive knowledge. Since EMNEs are not present as long as DMNEs in developed markets, applicants from these markets usually have less knowledge about EMNEs than about DMNEs (Held & Bader, 2018). This lack of knowledge will motivate them to look for more information about EMNEs. Specifically, applicants in developed markets would be more concerned about CSR information of EMNEs which compensates for the prevalent institutional voids in the home countries. In contrast, when assessing MNEs originated from similar strong institutional environment (DMNEs), they can be assured that responsible social conducts have been institutionalized (Mazboudi et al., 2020), hence searching for additional CSR information is less necessary. Therefore, we hypothesize:

*Hypothesis 2*: Emerging country-of-origin of an MNE (vs. developed country-of-origin) increases the willingness of potential applicants to search for CSR information of the MNE.

Mainly drawing upon signaling and social-identity theory, scholars have extensively studied the effects of CSR and found that CSR increases applicants’ perception of organizational attractiveness and job-pursuit intention, (Backhaus et al., 2002; Behrend et al., 2009; Evans & Davis, 2011; Greening & Turban, 2000; Jones et al., 2014; Kim & Park, 2011; Rupp et al., 2013). According to Jones et al. (2014), CSR influences applicants’ job-pursuit intention through three signal-based mechanisms: expected treatment or working environment, person-organization fit, and anticipated pride from being associated with the organization. Social-identity theory (Ashforth & Mael, 1989), in addition, suggests that self-image of an employee is influenced by his or her membership in an organization. Thus, it is reasonable to infer that applicants are more likely to pursue a job with companies engaging in CSR as it contributes to their positive self-image (Greening & Turban, 2000). By extending these findings to the context of international recruitment, local applicants may have limited information about foreign MNEs as compared to domestic companies. Thus, MNEs can engage in CSR to signal a good working environment, thereby increasing their attractiveness as a potential employer to local applicants. Yet, it remains unknown whether CSR signals are evaluated differently based on the firm’s country-of-origin. Therefore, the interaction between CSR and a macro-level factor – country-of-origin of MNEs is of greater interest of this study. Combining reputation theory from economics with role theory from sociology, Jensen et al. (2012) argue that reputation and reputational signals become most salient when it is incongruent with role expectations. In other words, identical signals may have different effects for different actors. We, therefore, expect CSR to have great value for EMNEs to mitigate the negative effect of unfavorable country-of-origin image on job-pursuit intention. It gives them a positive image element and provides a company characteristic that applicants may otherwise not expect from an EMNE. In contrast, DMNEs already benefit from a more favorable country-of-origin image, so CSR signaling is likely to provide little additional information, resulting in a weaker positive effect on applicants’ job-pursuit intention. Thus, we assume that:

*Hypothesis 3*: CSR perception weakens the negative relationship between emerging country-of-origin of an MNE and job-pursuit intention.

### Situational Skepticism

Even though researchers have found that CSR is a means to strengthen legitimacy and to attract local talents, MNEs might run the risk involved in communicating about their CSR activities, which is stakeholders’ skepticism. This CSR dilemma has been clearly depicted in studies of Bachmann and Ingenhoff (2016) and Du et al. (2010). On the one hand, stakeholders demand to know more about good acts of companies, but on the other hand they tend to become suspicious of the sincerity of the CSR motives when the companies extensively promote their CSR efforts. According to social psychologists, people have a tendency to care more about why others do things than about what they do (Donia et al., 2019; Joo et al., 2016). Particularly, CSR can backfire if there is much situational skepticism (Forehand & Grier, 2003; Yoon et al., 2006). Attribution theory provides a suitable framework for a situation-based analysis of stakeholder skepticism. Based on attribution theory, when reading about the firm CSR initiatives, individuals would be likely to initially suspect the motives behind those initiatives and thus process the given information systematically to find out true motives of the firm. If they attribute the firm CSR activities to low sincere motives, it will result in higher skepticism, which in turn negatively influences subsequent attitudes and behaviors (Du et al., 2007; Forehand & Grier, 2003; Yoon et al., 2006). By extending these findings to the context of international recruitment, it is reasonable to infer that CSR perception can only lead to positive inferences about MNEs, and hence increasing organizational attractiveness when high sincere motives are attributed. Scholars have found evidence that stakeholders’ previous perception of firms influences the motive attribution process of the firms’ CSR (Du et al., 2010; Elving, 2013; Kim & Lee, 2012; Yoon et al., 2006). Elving (2013) explains that for firms with good reputation, people will not doubt the firms’ motives. In fact, they may not even consider the firm’s motives at all, which scales back the effects of sincere motive attribution. Meanwhile, for a firm with bad reputation, people will be more sensitive and engage more in the causal attribution, hence amplifying the effect of motive attribution. Especially, Vidaver-Cohen et al. (2015) document that country-of-origin affects reputation perception of MNEs. They have found that DMNEs from United States and Northern Europe received higher ratings on reputation than EMNEs from Latin America and Southern Europe. This is also in line with a review of corporate reputation by Mitra et al. (2013). These researchers find that EMNEs suffer bad corporate reputation from their country-of-origin, which are exemplified through broad lenses of deficiency in sociohistorical specificities, instability, corruption, outlandish culture customs, and strict authoritarianism. Referring to these findings, we expect that CSR perception only increases EMNEs’ organizational attractiveness and weaken the negative relationship between emerging country-of-origin and job-pursuit intention when applicants attribute sincere motives. Our assumption is also strengthened by the paradox of double-edge legitimacy proposed by Ashforth and Gibbs (1990) such that publics are more skeptical of legitimation attempts of firms with lower perceived legitimacy. Unlike DMNEs, EMNEs suffer from double disadvantages of liabilities of foreignness plus liabilities of origin from a developing economy home base, resulting in lower legitimacy for EMNEs, as compared to DMNEs (Contractor, 2013; Held & Bader, 2018). Hence, if EMNEs try to engage in CSR to make up for their tarnished country-of-origin image and gain legitimacy, applicants may perceive their CSR efforts as insincere, which reduces the positive effect of CSR. We, therefore, propose that:

*Hypothesis 4*: The moderating effect of CSR perception on the negative relationship between emerging country-of-origin and job-pursuit intention is stronger when sincere motive attribution is high.

**Fig. 1:**
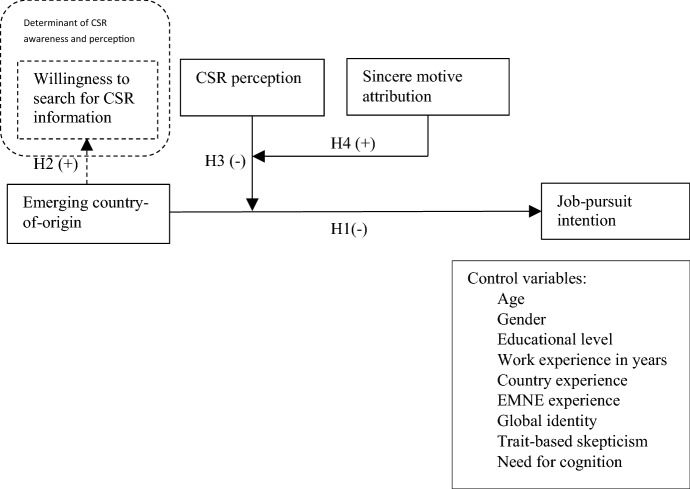
Conceptual model

The conceptual framework is illustrated in Fig. 1.

## Methodology

### Experimental Design

Our study is based on a 2 (country of origin: 5 developed vs. 5 emerging) × 3 (CSR: good vs. mediocre vs. poor) between-subjects experimental design. We used several developed and several emerging markets as stimuli to reduce any idiosyncratic country image influence beyond the distinction in developed vs. emerging. For this, we identified 5 emerging and 5 developed countries that were, in the context of recruitment, homogeneous within and heterogeneous between. Thirty fictitious corporation career webpages were created. Following the approach of Hong and Kim (2017), we selected a fictitious foreign medium-sized consumer goods manufacturer to preclude any potential emotional ties of respondents with an existing company. Moreover, when respondents are unfamiliar with the company, they will rely more on the available signals from country-of-origin or CSR in their decision-making process (Han, 1989). The reason for selecting consumer goods sector is that it is highly visible to people and accounts for 35% of material inputs used globally and 75% of municipal solid waste, which has a high polluting potential and is subject to high pressure and scrutiny from stakeholders (EMF, 2013; González-Benito & González-Benito, 2006). To enhance the external validity, the construction of the webpage is based on career pages of real companies. We selected Mars, Inc. and SC Johnson as examples because they are consumer goods manufacturers that appeared in the list of the World’s 20 Best Workplace 2018 – Multinational (Great-Place-to-Work, 2018) and both have international operations in Switzerland, Germany, and Austria. The job opportunities offered are junior manager positions. The logic behind this is that the junior management level is attractive to our participants, and thus motivate them to navigate throughout the webpage. Each webpage was available in both English and German. Screenshots of the webpages are provided in Appendix A.

For recognition of country-of-origin and CSR, all webpages included information on the country-of-origin and CSR initiatives. CSR activities, based on Kinder, Lydenberg, Domini (KLD) ratings, consist of 11 dimensions. Of these dimensions, the five dimensions environment, community relations, diversity, product issues, and employee relations are more important than the others and have been widely used in prior research. Especially, Backhaus et al. (2002) find that these five have the largest effects on firm attractiveness as an employer. The good CSR condition described what firms are currently doing or have done with concrete indicators, such as resource allocation and actual impacts related to each of the five CSR dimensions. The mediocre CSR condition only showed statements and intentions that do not necessarily translate into real actions. In the poor CSR condition, we mentioned some but not all the five CSR dimensions with only a few superficial sentences. Four business lecturers from two universities carefully reviewed the three scenarios and agreed that they adequately portrayed different levels.

### Pretest for Country Image

To identify five homogeneous emerging and five homogeneous developed countries in the context of recruitment, a pretest was conducted on the respondent perception of countries/territories, starting with a list of 10 developed and 10 emerging markets. The selection of countries/territories is based on the latest Human Development Index (HDI) of the United Nations (UNDP, 2019). The developed group contains the ten highest ranking countries/territories with regard to HDI, excluding Switzerland (2nd) and Germany (4th) because the study respondents are from these two countries. Thus, the ten countries/territories are Norway, Australia, Hong Kong, Sweden, Singapore, Netherlands, Denmark, Finland, Canada, and US. These selected countries/territories are classified as developed markets by FTSE (2019) and are among the top 25 of the World Competitiveness Ranking by World Economic Forum (Schwab, 2019). On the other hand, the emerging group consists of Asian emerging countries/territories in the range of middle and high index value. We chose to focus in this region as it is one of main drivers of the world economy according to IMF (Lagarde, 2016). They are China, Taiwan, India, Sri Lanka, Indonesia, Philippines, Malaysia, Vietnam, Thailand, and Bangladesh. These selected countries/territories represent a range in terms of the size of population, economic development, religious affiliation, and political experience, which could be representative of Asian emerging markets.

**Fig. 2:**
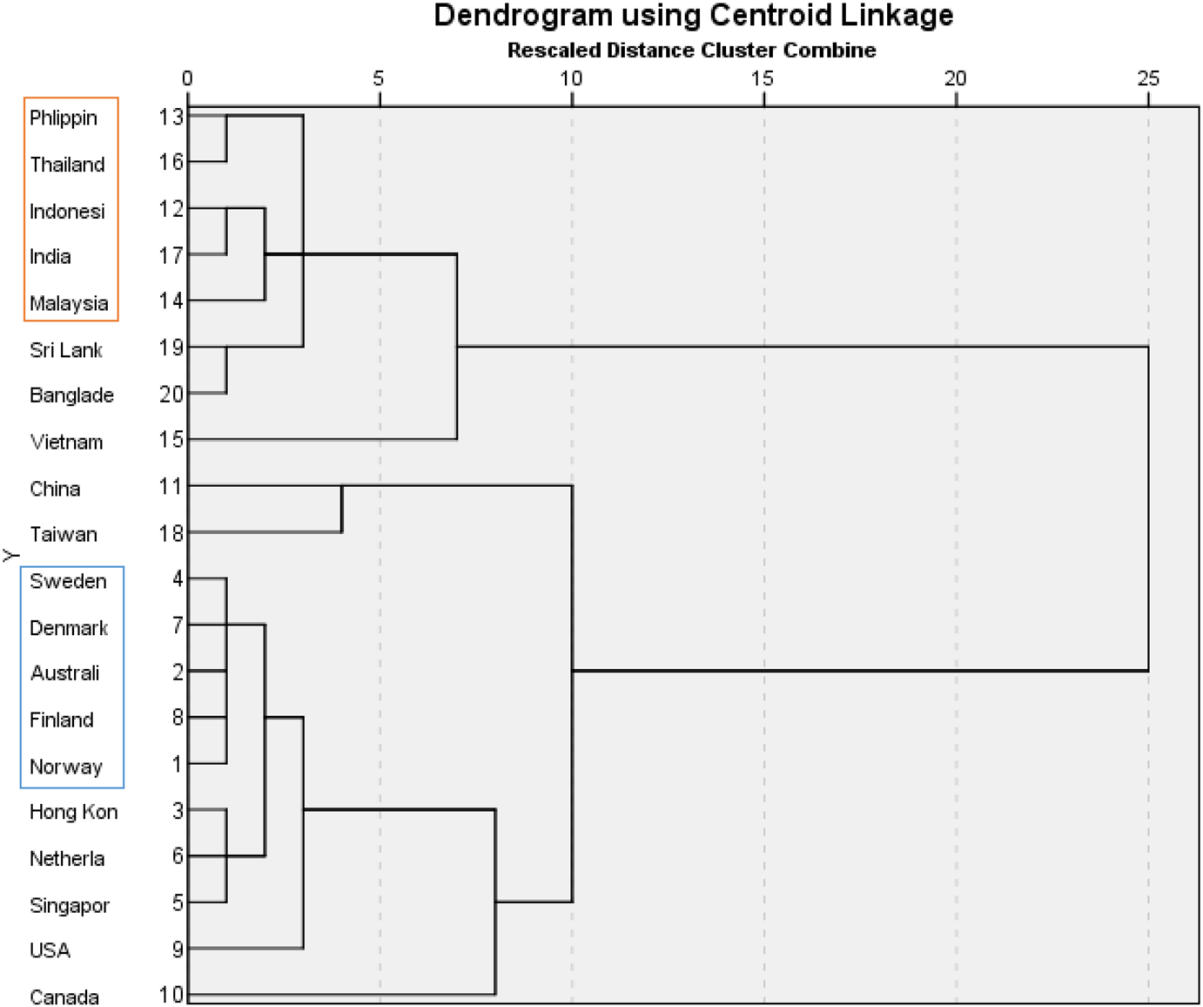
Cluster analysis result in the pretest

The purpose of the pretest was to find five markets in each group with high homogeneity within one group and significant heterogeneity across the two groups in the aspect of country-of-origin image in the context of recruitment. To do that we first needed to operationalize the concept of country-of-origin image which we did based on the three-dimensional construct of Froese et al. (2010) for the specific context of recruitment. We administered our pretest surveys to 76 participants, who were randomly assigned to one of the 5 questionnaire versions, each containing questions on four countries/territories (2 developed and 2 emerging). Through factor analysis of the seven items we obtained two factors (see Table 1). The first includes items representing the cognitive perception of technological development and general human resource practice of a country (α = 0.879). The second includes items representing the affective perception of in-group favoritism of people from a country (α = 0.710). From these two factors, the countries were segmented using the hierarchical cluster method, which is widely used in the literature for small samples (Janssens et al., 2008). Applying this method, the selection of two groups proved to be ideal, namely the developed country group: Sweden, Denmark, Australia, Finland, Norway and the emerging country group: Philippines, Thailand, Indonesia, India, Malaysia (see dendrogram in Fig. 2).

**Table 1:** Factor analysis of country-of-origin image in the pretest

	1	2
Working for a company from [Country name] provides good opportunities for career advancement	**0.932**	0.054
Working for a company from [Country name] provides good training	**0.895**	− 0.034
[Country name] is a technologically and scientifically advanced country	**0.860**	0.155
A job at a company from [Country name] would have a good working environment	**0.740**	− 0.291
[Country name] people discriminate against non-[Country name] people	− 0.037	**0.849**
[Country name] people are arrogant	0.068	**0.767**
[Country name] people socialize only with their own people	− 0.069	**0.753**

Bold values indicate the significance results

One interesting finding from our pretest is that China and Taiwan are categorized as a separate cluster from the other emerging markets and eventually even grouped with the developed markets. These two emerging markets are catching up with developed nations in terms of technological development and career prospects. Their competitive advantage is no longer predicated solely upon low-cost labor (Devang et al., 2017).

### Sample

Third-year bachelor and master students majoring in business at several universities in Germany, Switzerland and Austria are the main focus of the study because they will soon graduate, and graduates constitute a major part of the future qualified labor force for MNEs operating in these countries. The sample exclusively consists of German, Swiss and Austrian citizens to eliminate any potential different perceptions caused by other nationalities. Since these three countries are German-speaking countries, the questionnaire was translated into German. To ensure the reliability, all items were translated from English into German by a bilingual researcher and back-translated into English by another bilingual researcher (Mullen, 1995). 589 students took part in the study voluntarily, and 257 completed it. We also recruited additional 318 participants via a crowdsourcing platform. Researchers have considered crowdsourcing platforms a useful and cost-effective source of data for research in the behavioral sciences (Goodman et al., 2013; Simcox & Fiez, 2014). There is growing prevalence of crowdsourcing literature: more than 40% of the studies published in the Journal of Consumer Research issue 42 used crowdsourcing platforms for data collection (Goodman & Paolacci, 2017; Mellis & Bickel, 2020). We implemented one validity-check question and time limit options in the survey to control for the quality of the responses collected via the platform, consistent with the best practices suggested by Cobanoglu et al. (2021). The demographic information of these participants is shown in Table 2. We compared the two sample groups using Kolmogorov–Smirnov tests and the results show that they are comparable in all demographic characteristics (*p* > 0.05), except for age. The crowdsourcing sample was younger than our original university sample. Nevertheless, our target group aged between 20 and 30 remained dominant in both samples. We compared the responses of the two groups further by conducting t-tests on the key variables. The results show that for none of our key variables, the two groups display a significant difference (CSR perception: *M*_university_ = 4.49, *M*_crowdsourcing_ = 4.70, *t*(326) =  − 1.64, *p* > 0.05; sincere motive attribution: *M*_university_ = 4.57, *M*_crowdsourcing_ = 4.82, *t*(326) =  − 1.88, *p* > 0.05; job-pursuit intention: *M*_university_ = 4.49, *M*_crowdsourcing_ = 4.78, *t*(326) = − 1.88, *p* > 0.05).

**Table 2:** Sample characteristics

	Reduced set n = 328 (%)	Final sample n = 490 (%)	Total sample n = 575 (%)	Crowdsourcing sample n = 318 (%)	University sample n = 257 (%)
Gender					
Male	46	47.9	49	54.1	42.6
Female	54	52.1	51	45.9	57.4
Age					
Less than 20	7.9	6.9	7	7.5	6.2
20–30	86.9	88	88	91.6	83.7
Over 30	5.2	5.1	5	0.9	10.1
Education					
Less than Bachelor’s degree	58.5	54.7	53.7	56.3	50.6
Bachelor’s degree	32	34.9	35.3	34	37
Master’s degree	8.6	9.8	10.3	9.4	11.3
Doctorate	0.9	0.6	0.7	0.3	1.1
Country experience					
Yes	84.5	15.9	16.7	17.6	15.6
No	15.5	84.1	83.3	82.4	84.4
EMNE work experience					
Yes	6.4	5.9	7.3	7.2	7.4
No	93.6	94.1	92.7	92.8	92.6
EMNE visit experience					
Yes	11.3	12	13.2	12.3	14.4
No	88.7	88	86.8	87.7	85.6

From those 575, we excluded 85 participants who spent a very short time on the company webpages. Our final sample consists of 490 participants (282 German, 111 Swiss, and 97 Austrian). Their average age was 24.24 years (SD = 3.86), and 34% were actually looking for a job. We further excluded 162 participants from the hypotheses tests related to CSR who did not open the sub-page “Sustainability” as these participants were not exposed to our CSR manipulations. We used Kolmogorov–Smirnov tests to statistically test whether those remaining form a good representation of the total set. The results indicate that both the final and reduced samples are comparable to the total sample for all control demographic variables (*p* > 0.05).

### Procedure

Participants were randomly assigned to one of 30 scenarios. At first, participants were asked to imagine themselves as applicants who had found a MNE that was currently recruiting junior managers. Then, they were asked to click on the link of the career page of that MNE to get more information. On the career page, we provided the same brief description of the company, open positions offered, salary level and compensation package obtained from real recruitment advertisements, in order to eliminate any potential effects of extraneous factors on job-pursuit intention. Then, participants could further access an “About us” section giving in each scenario the same description of the MNE as a global player, except for the country-of-origin. There was also a “Sustainability” section revealing different CSR activities depending on specific scenarios. Afterwards, participants answered questions on manipulation checks, their attraction to the organization, motive attribution, personal traits, the relative importance of five CSR dimensions in their job-choice process, and demographic characteristics.

### Measures

#### Willingness to Search for CSR Information

This variable was measured by whether a respondent opened the sub-page “Sustainability” from the MNE’s career page using Google Analytics and User Activity Tracking plugin. With these tools, we can also explore viewing time, page-view order, and bounce rate (percentage of all sessions in which users view only a single page then exit without opening others).

#### CSR Perception (α = 0.74)

Following (Joo et al., 2016), four items were developed to reflect respondents’ perceptions of the MNE’s CSR activities: “This company has placed significant efforts to improve energy efficiency in its daily operation,” “This company implements programs to improve its products for the well-being of its customers,” “This company offers training to its employees and establishes measures to actively support employment and fair treatment toward women and minority employees,” and “This company made donations to improve the well-being of society.”

#### Sincere Motive Attribution (α = 0.89)

We used three items adapted from Du et al. (2007); Yoon et al. (2006). A sample item is “This company has genuine concerns for the environment, customers, employees, and community.”

#### Job-Pursuit Intention (α = 0.90)

We employed Highhouse et al. (2003)’s measure of job-pursuit intention. A sample item is “I would make this company one of my first choices as an employer.”

All the above-mentioned constructs were measured using a 7-point Likert scale anchored from 1 (strongly disagree) to 7 (strongly agree) for a coherent structure throughout the survey.

### Manipulation Checks

Manipulation checks for web page realism, participant engagement and their perceptions of the MNE’s country-of-origin and CSR activities were carried out to ensure the validity of the experiment. Following Jones et al. (2014), we check whether participants perceived the web page to be realistic by one item: “The information from the company’s web page looked like it was from the real web page” (*M* = 5.26, SD = 1.40) and whether they engaged in their roles as applicants by two items: “I really tried to imagine that I was looking for a job” (*M* = 5.84, SD = 1.24) and “If I were actually looking for a job, I’d like to read information from company websites like I did in this study” (*M* = 5.85, SD = 1.34). Respondents perceived the website to be realistic and were engaged in the applicant role as the mean values on a 7-point agreement scale all exceed 5.25.

Then, participants were asked to assess the MNE’s country-of-origin as well as its CSR activities. The t-tests results show that the developed country group was perceived to have higher technological development and better general human resource practice than emerging country group (*M*_developed_ = 5.42, *M*_emerging_ = 4.10, *t*(488) = 15.39, *p* < 0.001), whereas there is no significant difference in the perception of in-group favoritism of people from developed country and emerging country groups (*M*_developed_ = 2.14, *M*_emerging_ = 2.17, *t*(488) = 0.34, *p* > 0.05).

Regarding CSR perception, a one-way ANOVA showed that values are significantly different in the good, mediocre, and poor CSR conditions (*M*_good_ = 5.27, *M*_mediocre_ = 4.48, *M*_poor_ = 3.91, *F*(2, 325) = 32.02, *p* < 0.001, *η*^2^ = 0.17). Results of post hoc tests indicated that participants who were assigned to the good CSR manipulation had higher CSR perception than those assigned to the mediocre CSR condition (95% CI [0.46, 1.11], *p* < 0.001) and to the poor condition (95% CI [1.01, 1.71], *p* < 0.001). In addition, participants in the mediocre CSR condition had higher CSR perception than those in the poor CSR condition (95% CI [0.23, 0.93], *p* < 0.001). In addition, we ran one-way ANOVA separately for DMNEs and EMNEs and found that those significant differences across the three CSR conditions remained for both DMNEs and EMNEs.

Overall, these results show that the manipulations worked as intended and created significant variance in the independent variables.

### Control Variables

To exclude confounding effects of demographic variables as in prior studies assessing job-pursuit intention, we controlled for respondent age, gender, education level, working experience, country experience, EMNE experience, trait-based skepticism, need for cognition, and global identity.

## Results

### Measurement Equivalence

Since we collected cross-national data, our measures need to exhibit an adequate equivalence across cultures. Following the procedures recommended by Vandenberg and Lance (2000), we conducted multigroup confirmatory factor analysis (CFA) for each of our measures across the three culture groups using AMOS 26. Each measure was examined separately because separating CFA tests helps reduce the likelihood that extraneous covariances cloud the understanding of individual item functioning (Rupp et al., 2018). Table 3 presents the model fit indices from these CFA tests. As can be seen from these results, the model assessing metric invariance fits the data well relative to the model assessing configural invariance (insignificant chi-square differences), which supports measurement equivalence in our study. Thus, the pooling of the three national samples for subsequent hypothesis testing is justified.

**Table 3:** Measurement equivalence tests for study variables

Variables	χ^2^	*df*	CFI	GFI	RMSEA	Δχ^2^	Δ*df*	*p*
Job-pursuit intention								
Configural invariance	56.78	15	.97	.95	.08	10.14	8	*p* > .05
Metric invariance	66.92	23	.97	.95	.06			
Country image								
Cognitive								
Configural invariance	10.59	6	.99	.99	.03	6.67	6	*p* > .05
Metric invariance	17.26	12	.99	.99	.03			
Affective								
Configural invariance	0.00	0	1.00	1.00	.03	6.06	4	*p* > .05
Metric invariance	6.06	4	1.00	.99				
CSR perception								
Configural invariance	22.27	6	.97	.98	.07	6.47	6	*p* > .05
Metric invariance	28.74	12	.97	.98	.05			
Sincere motive attribution								
Configural invariance	0.00	0	1.00	1.00	.06	9.34	4	*p* > .05
Metric invariance	9.34	4	.99	.99				
Trait-based skepticism								
Configural invariance	0.00	0	1.00	1.00	.00	2.49	4	*p* > .05
Metric invariance	2.49	4	1.00	1.00				
Need for cognition								
Configural invariance	0.00	0	1.00	1.00	.00	3.72	4	*p* > .05
Metric invariance	3.72	4	1.00	1.00				
Global identity								
Configural invariance	11.80	6	.99	.99	.04	6.13	6	*p* > .05
Metric invariance	17.93	12	.99	.99	.03			

*CFI* comparative fit index; *GFI* goodness of fit index; *RMSEA* root‐mean‐square error of approximation

### Common-Method Variance

Common-method variance (CMV), which is defined in terms of variance attributable to the measurement method rather than to constructs of interest, poses a serious threat for bias in social science research, particularly with a single time and single source survey (Podsakoff et al., 2012). Since our study is based on a single time survey in which both independent and dependent variables were collected from the same respondents with similar response scales, we need to control for the presence of CMV and examine whether it caused any systematic error. Nevertheless, it should be noted that in an ongoing debate on the problems with CMV, a recent study of Fuller et al. (2016) has empirically proved that CMV can bias results *only* when CMV is present at a relatively high level (approaching 70% or more) and that these cases are indeed not common in practice.

Following Podsakoff et al. (2012), we controlled for the bias by both ex-ante procedures in the questionnaire design and ex-post statistical checks. Some ex-ante procedural remedies applied were assuring respondent anonymity and confidentiality, emphasizing that there were no right or wrong answers to reduce evaluation apprehension, improving item wording to avoid ambiguity, and varying order of items. We also addressed ex-post statistical remedies using Harman's single-factor test. Although one may question the reliability of this test, Fuller et al. (2016) have demonstrated that criticisms of the Harman test’s reliability are partially unfounded. Specifically, they found that false negatives (i.e., the Harman test indicates no common-method bias when the bias is present) are much less common than false positives (i.e., the test indicates bias from CMV when none is evident in data). The false positives may occur in high-reliability datasets with Cronbach's *α* higher than 0.95, which is not the case in our study. The Harman test’s unrotated factor solution revealed that a seven-factor model explained 66.47% of the total variance and no single factor accounted for more than 50% of the variance (the highest was 27.63%). Since no single factor emerged to account for the majority of covariance among variables, it appears that CMV is not a major concern. We also applied a CFA approach to Harman's one-factor analysis and compared the one-factor Harman's confirmatory factor analysis solution to the seven-factor solution. We found that the single-factor solution did not fit the data well (χ^2^_(324)_ = 3124.42, *p* < 0.01, CFI = 0.63, TLI = 0.59, RMSEA = 0.13) and was indeed significantly worse than the seven-factor solution (△χ^2^_(21)_ = 2605.31, *p* < 0.01). To gauge the extent of CMV, we additionally included an unmeasured common latent factor in CFA. This common latent factor did not account for substantial variance in the indicator variables (only 2.25%). Furthermore, we found no changes in the path directions and significances between the constrained (factor loadings are constrained to zero) and unconstrained (factor loadings are estimated freely) models, implying that a single method-driven factor did not represent our data (Lowry et al., 2013). Overall, these statistical tests provide adequate support that CMV does not pose a significant threat to this study.

### Preliminary Checks

As a preliminary check of our basic premise, we investigated the relative importance of CSR dimensions to Swiss, German, and Austrian applicants in their job-choice process. As expected, we found that applicants considered all five CSR dimensions important (*M* = 5.62, SD = 0.86). Overall, applicants consider CSR dimension of employee relations the most important (71% males and 82% females rated this dimension as very important/important, 6–7 rating on the 7-point scale), followed by product issues and environment. The least important is the dimension of community relations. Females place more importance on all five CSR dimensions than males, and in particular on the diversity dimension (72% females versus 38% males rated diversity important).

In line with our research objective to address applicants’ awareness of firm CSR activity, we investigated their behaviours on the corporate webpage. In general, page-view order is the same as the page order in the navigation menu. Consequently, when the “Sustainability” page was located at the bottom of the navigation menu, it reduces the probability of being viewed for that page. We did not find a large difference in website viewing time between DMNEs and EMNEs (134 versus 132 s). However, as expected, the bounce rate of DMNEs’ websites (20%) was higher than that of EMNEs’ websites (14%), which shows that applicants were more motivated to process further information about EMNEs. We will return to this in the next section.

### Hypotheses Tests

Table 4 presents the correlation matrix. Consistent with our expectations, job-pursuit intention negatively correlated with emerging country-of-origin and positively correlated with CSR perception.

**Table 4:** Correlation matrix

	1	2	3	4	5	6	7	8	9	10	11	12	13	14
1. Age	–													
2. Gender	− 0.139^*^	–												
3. Education	0.390^**^	0.020	–											
4. Working experience	0.642^**^	− 0.094	0.116^*^	–										
5. EMNE work experience	0.172^**^	0.042	0.260^**^	0.257^**^	–									
6. EMNE visit experience	0.048	0.039	0.189^**^	0.133^*^	0.497^**^	–								
7. Country experience	0.101	0.160^**^	0.134^*^	0.108^*^	0.128^*^	0.113^*^	–							
8. Trait-based skepticism	0.080	− 0.078	− 0.002	0.023	0.014	− 0.035	− 0.022	–						
9. Need for cognition	− 0.021	0.053	0.124^*^	− 0.020	− 0.040	0.031	0.099	− 0.023	–					
10. Global identity	0.000	0.142^*^	− 0.055	0.036	0.034	0.099	0.003	− 0.099	0.187^**^	–				
11. Country-of-origin	− 0.005	− 0.056	0.010	− 0.016	− 0.012	0.016	− 0.251^**^	0.018	− 0.010	− 0.034	–			
12. CSR perception	− 0.041	0.133^*^	− 0.070	− 0.007	− 0.152^**^	− 0.082	− 0.028	− 0.189^**^	− 0.024	0.092	− 0.045	–		
13. Sincere motive attribution	− 0.101	0.142^*^	− 0.133^*^	− 0.077	− 0.127^*^	− 0.108	− 0.049	− 0.266^**^	− 0.028	0.175^**^	− 0.143^**^	0.592^**^	–	
14. Job-pursuit intention	0.020	0.146^**^	− 0.050	− 0.029	− 0.049	0.012	0.054	− 0.226^**^	− 0.033	0.232^**^	− 0.248^**^	0.415^**^	0.573^**^	–

N = 328. Gender was coded as 0 = male, 1 = female; Educational level was coded as 0 = less than Bachelor’s degree, 1 = at least Bachelor’s degree; EMNE work experience/EMNE visit experience/Country experience were coded as 0 = no, 1 = yes; Country-of-origin was coded as 0 = developed, 1 = emerging

^+^*p* < 0.10, *p < 0.05, **p < 0.01, ***p < 0.001

To test our hypotheses, we conducted hierarchical moderated regression. We centered all continuous predictor variables before entering them into the regression equation and generating the interaction terms, following the approach of Aguinis (2004) and Rupp et al. (2018). We first entered all control variables (i.e., respondent age, gender, education level, working experience, country experience, EMNE experience, trait-based skepticism, need for cognition, and global identity) into the regression equation. In the second and third steps, we entered the main effects of country-of-origin, CSR perceptions, sincere motive attribution, and the two-way interactions among them, respectively. In the last step, we entered the three-way interaction of country-of-origin, CSR perceptions, and sincere motive attribution. The results (see Table 5) revealed that originating from emerging markets reduces job-pursuit intention (*β* = -0.15, *p* < 0.01). The t-test results converged with our regression analyses: job-pursuit intention (*M*_developed_ = 4.96, *M*_emerging_ = 4.33, *t*(326) = 4.69, *p* < 0.001). Thus, Hypothesis 1 is supported.

**Table 5:** Regression results: Hierarchical moderated regression analyses of job-pursuit intention

Variables	Step 1			Step 2			Step 3			Step 4		
	B	SE	β	B	SE	β	B	SE	β	B	SE	β
	3.49	0.55		3.71	0.46		3.75	0.46		3.76	0.46	
Age	0.05	0.02	0.14	0.05	0.02	0.15^*^	0.05	0.02	0.14*	0.04	0.02	0.14^*^
Gender	0.28	0.14	0.11^*^	0.11	0.12	0.04	0.08	0.11	0.03	0.08	0.12	0.03
Education	− 0.17	0.16	− 0.06	− 0.06	0.13	− 0.02	− 0.05	0.13	− 0.02	− 0.04	0.13	− 0.02
Working experience	− 0.05	0.04	− 0.10	− 0.05	0.03	− 0.10	− 0.04	0.03	− 0.09	− 0.04	0.03	− 0.08
EMNE work experience	− 0.31	0.32	− 0.06	− 0.09	0.27	− 0.02	− 0.10	0.27	− 0.02	− 0.09	0.27	− 0.02
EMNE visit experience	0.10	0.24	0.02	0.28	0.20	0.07	0.29	0.20	0.07	0.29	0.20	0.07
Country experience	0.19	0.19	0.06	0.10	0.16	0.03	0.15	0.16	0.04	0.14	0.16	0.04
Trait-based skepticism	− 0.21	0.06	− 0.20^***^	− 0.08	0.05	− 0.07	− 0.07	0.05	− 0.07	− 0.07	0.05	− 0.07
Need for cognition	− 0.16	0.06	− 0.14^**^	− 0.10	0.05	− 0.09^*^	− 0.10	0.05	− 0.10^*^	− 0.11	0.05	− 0.10^*^
Global identity	0.25	0.06	0.22^***^	0.16	0.05	0.14^**^	0.17	0.05	0.15^**^	0.17	0.05	0.15^**^
Country-of-origin (Emerging Market)				− 0.43	0.11	− 0.17^***^	− 0.44	0.11	− 0.17^***^	− 0.38	0.13	− 0.15^**^
CSR perception				0.13	0.06	0.12^*^	− 0.04	0.09	− 0.04	− 0.07	0.09	− 0.06
Sincere motive attribution				0.43	0.06	0.43***	0.49	0.09	0.49^***^	0.52	0.09	0.51^***^
Country-of-origin x CSR							0.32	0.12	0.22^**^	0.34	0.12	0.23^**^
Country-of-origin x sincere motive attribution							− 0.10	0.11	− 0.08	− 0.13	0.12	− 0.10
CSR x sincere motive attribution							− 0.01	0.03	− 0.01	0.03	0.05	0.05
Country-of-origin x CSR x sincere motive attribution										− 0.07	0.07	− 0.08
*F*	5.29^***^			17.07^***^			14.72^***^			13.91^***^		
*R*^*2*^	0.14			0.41			0.43			0.43		
Adjusted *R*^*2*^	0.12			0.39			0.40			0.40		
ΔR^2^				0.27^***^			0.02^*^			0.00		

N = 328. Gender was coded as 0 = male, 1 = female; Educational level was coded as 0 = less than Bachelor’s degree, 1 = at least Bachelor’s degree; EMNE work experience/EMNE visit experience/Country experience were coded as 0 = no, 1 = yes; Country-of-origin was coded as 0 = developed, 1 = emerging

^+^*p* < 0.10, *p < 0.05, **p < 0.01, ***p < 0.001

Tracking applicants’ behaviours on the corporate webpage, we found that they were more willing to search for CSR information for EMNEs than for DMNEs, supporting Hypothesis 2. Specifically, the percentages of applicants that clicked open the sub-page “Sustainability” for EMNEs and DMNEs were 73.66% and 60.32% (χ2 = 9.85, *df* = 1, *p* < 0.01), respectively.

**Fig. 3:**
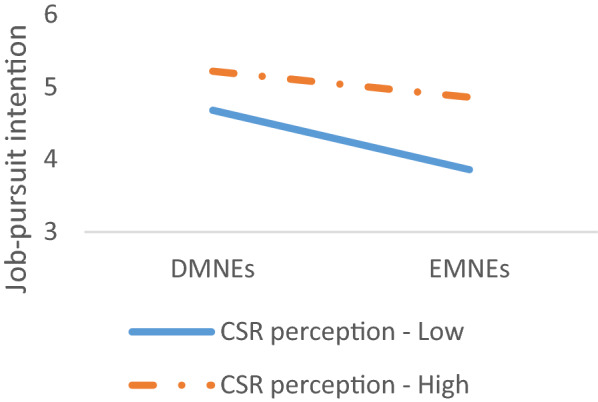
Two-way interaction between country-of-origin and CSR perception on job-pursuit intention

The two-way interaction between emerging country-of-origin and applicants’ CSR perception on job-pursuit intention was significant (*β* = 0.23, *p* < 0.01). Figure 3 presents the two-way interaction. Simple slopes analyses and slope difference test revealed that CSR perception diminished the negative relationship between emerging country-of-origin and job-pursuit intention such that the relationship was weaker when CSR perception was high (*B* = -0.27*, p* > 0.05) versus low (*B* = -0.91*, p* < 0.001; slope difference = 0.64, *t* = 2.61, *p* < 0.01). Therefore, Hypothesis 3 is supported.

**Fig. 4:**
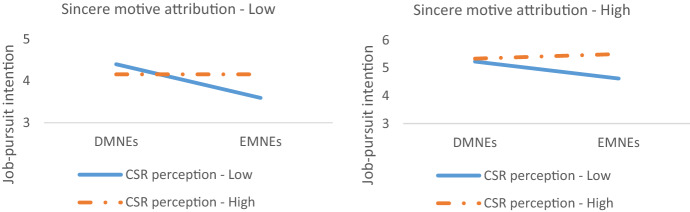
Three-way interaction of country-of-origin, CSR perception, and sincere motive attribution on job-pursuit intention

Although emerging country-of-origin is negatively related to sincere motive attribution as expected (*correlation* = -0.14, *p* < 0.01), we did not find a significant three-way interaction of country-of-origin, CSR perception, and sincere motive attribution on job-pursuit intention (*β* = -0.08, *p* > 0.1). Consequently, Hypothesis 4 is not supported. The effect of CSR perception surpassed our expectation. High level of perceived CSR increases job-pursuit intention for EMNEs, which weakens the negative effect associated with emerging country-of-origin regardless of motive attribution (see Fig. 4). Despite the insignificant three-way interaction, sincere motive attribution still plays a direct role in influencing applicants’ job-pursuit intention (*β* = 0.51, *p* < 0.001).

### Supplementary Analysis

**Fig. 5:**
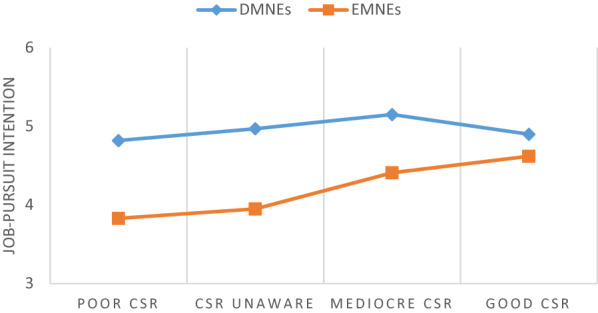
Effects of country-of-origin and CSR activities on job-pursuit intention

We conducted ANOVA to evaluate the differences in job-pursuit intention between CSR aware group (those who opened the sub-page “Sustainability”) and CSR unaware group (those who did not) with separation between EMNEs and DMNEs (see Table 6 and Fig. 5). The results indicated that for EMNEs, job-pursuit intention was significantly higher when applicants were aware of the company’s good CSR as compared to when they were aware of the company’s poor CSR and when they were unaware of the company’s CSR activities. On the other hand, the results for DMNEs showed no significant differences in applicants’ job-pursuit intention toward DMNEs whether they were unware or aware of the companys’ CSR (either good, mediocre or poor).

**Table 6:** Mean comparison of job-pursuit intention: results of ANOVA

	Mean				F value	Post-hoc test (Mean difference)					
	CSR Unware	CSR Aware				CSR unware vs. Poor CSR aware	CSR unware vs. Mediocre CSR aware	CSR unware vs. Good CSR aware	Poor vs. Mediocre CSR aware	Poor vs. Good CSR aware	Good vs. Mediocre CSR aware
		Poor	Mediocre	Good							
DMNEs, n = 247	4.97	4.82	5.15	4.90	0.87	0.15	− 0.18	0.07	− 0.33	− 0.08	− 0.25
EMNEs, n = 243	3.95	3.83	4.41	4.62	5.38^**^	0.12	− 0.46	− 0.67^*^	− 0.58^+^	− 0.79^**^	0.21

^+^*p* < 0.10, *p < 0.05, **p < 0.01, ***p < 0.001

## Discussion

In general, our findings indicate that the less favorable country-of-origin image of EMNEs in terms of career-enhancing job characteristics and technological development results in lower job-pursuit intention for EMNEs as compared to DMNEs. This confirms the expected liability of emergingness in international human resource management. It also substantiates previous findings (Held & Bader, 2018; Hong & Kim, 2017) that country-of-origin image presents an enormous challenge for EMNEs in attracting local talent in developed markets. The liability of emergingness is also confirmed by the finding that applicants are more skeptical about the sincerity of EMNEs’ CSR motives. Nevertheless, the mean differences are smaller than expected, suggesting that EMNEs are catching up with DMNEs in terms of attractiveness as potential employers in developed markets. There is also evidence in our pretest for the advancing of EMNEs: two emerging countries, China and Taiwan, were categorized in the same cluster as the developed countries.

Through the application of signaling and attribution theory, we argue that CSR helps mitigate the negative effect of originating from emerging countries on hiring talent in developed host countries. High levels of perceived CSR increase job-pursuit intention for EMNE even when applicants attribute low sincere motive to the firm’s CSR engagement. It could be explained that CSR is associated with great value to compensate for the prevalent institutional voids in home countries of EMNEs. Even if applicants consider the firm’s engagement as insincere, such engagement still benefits them and ensures a good employment opportunity. Unlike EMNEs, CSR is less influential for DMNEs. The less relevance of CSR for DMNEs was also shown by lower willingness to search for CSR information. In other words, CSR strategies can be an important factor influencing MNEs’ recruitment, particularly when the company’s country image is poor, in line with Mazboudi et al. (2020) and Hong and Kim (2017). Yet, one might question whether other traditional HR factors (i.e., salary) should be the central differentiator for EMNEs in attracting talent. Our results, of course, did not intend to undermine the importance of salary. However, we believe that for EMNEs, CSR stands a better chance of escaping the stereotypes of their own country-of-origin and staying competitive with DMNEs in the war for talent. Specifically, based on image and signaling theory, an applicant’s process of employer choice consists of two processes: (1) a screening process in which applicants absorb signals about different potential future employers and eliminate those that are not compatible with their values and principles from the pool of potential employers; (2) a choosing process in which applicants continue to examine the options left from the screening process and choose to pursuit job with the one offering the highest profitability to achieve their goals (Beach, 1993; Held & Bader, 2018; Spence, 1973). Therefore, it is imperative for EMNEs to firstly pass the compatibility test. Indeed, CSR has been found to enhance person-organization fit (Gully et al., 2013; Kim & Park, 2011; Zhang & Gowan, 2012). Aiman-Smith et al. (2001), moreover, have empirically shown that CSR (not salary) most strongly influenced the applicants’ attraction to an organization.

Further, although the three-way interaction between country-of-origin, CSR perception, and sincere motive attribution is insignificant, there is a significant positive direct relationship between sincere motive attribution and job-pursuit intention. It is evident, therefore, that sincere motive attribution still plays an important role in influencing applicants’ job-pursuit intention. Applicants do not only care about what the companies do, but also why they do, hence the importance of considering applicants’ skepticism.

We would also like to draw attention to unexpected findings about DMNEs. Firstly, when applicants attributed low sincere motive to the firm’s CSR engagement, high levels of CSR perception reduced applicants’ job-pursuit intention for DMNEs. Secondly, the mean values of job-pursuit intention were lower when applicants were aware of DMNEs’ good CSR as compared to when they were unware of the company’s CSR (despite insignificant mean differences). One explanation for that could be that applicants may have higher expectations for DMNEs. By studying the complexity of strategic CSR and corporate brand, Polonsky and Jevons (2009) indicate that MNEs, especially those from developed countries, need to address the highest set of global expectations since any lower level will be criticized for being less than appropriate. In our three CSR scenarios, only the good condition showed specific quantitative and monetary indicators demonstrating the firm’s actual impacts. Even though the provided figures are good, they might not meet the respondents’ expectations, particularly when they were not taken from companies with the best CSR reputation. This expectation is a reference point for evaluation (Cadotte et al., 1987; Han, 2015): when the firm’s CSR performance is lower than the expected level, information on CSR efforts backfire and the firm is evaluated negatively.

We emphasize the importance of applicants’ willingness to search for CSR information. Even though the CSR information is available on the corporate webpage, 33.13% of the potential applicants did not view it. Still, that means that two thirds of potential applicants search for such information. As a robustness check, we also calculated these percentages for only those respondents who were currently looking for a job and the results remain the same, supporting the validity of our finding. Moreover, excluding those that only viewed the first page and exited, we investigated those who opened other sub-pages, e.g. “About us”, but not the CSR sub-page. Interestingly, although they rated corporate social performance important (*M* = 5.39, SD = 0.91), they did not proactively seek CSR information, confirming previous findings of low public’s awareness of companies’ CSR activities. This is a serious impediment in firms’ attempts to maximize benefits from CSR engagement. It should be noted that the large percentage of applicants who did not look for CSR information could not undermine the importance of CSR as not looking does not necessarily equal not caring. As mentioned earlier, CSR is at the forefront on the global corporate agenda in today’s socially conscious market environment and there is convincing evidence of the importance of CSR from both academic research and marketplace polls. Our findings provide further evidence to support the importance of CSR. Specifically, CSR does matter and it is more a challenge for companies to make sure that the applicants really become aware of their CSR activities.

## Theoretical and Methodology Contributions

EMNEs’ applicant attraction in developed markets is an important yet under-researched area. This study investigates the effects of macro-level country-of-origin, firm-level CSR engagement and micro-level individual skepticism on job-pursuit intention for MNEs from five developed markets and five emerging markets, operating in Switzerland, Germany, and Austria. In doing so, our study, firstly, contributes to literature on the liability of emergingness (Held & Bader, 2018; Held & Berg, 2015), and aligns it with international human resource management, bringing both fields forward. Secondly, the study enriches CSR research on employee recruitment by adding a macro perspective (Maon et al., 2019), namely country-of-origin. Previous studies have mainly focused on individual-level variables (e.g., moral identity or personal relevance of CSR or socio-environmental consciousness) as contingency factors to explain the influence of CSR (Gond et al., 2017). Jones et al. (2019), nevertheless, have underlined that despite the fact that the vast majority of extant individual-focused CSR research is micro in nature, “the scientific study of these phenomena is by no means restricted to the individual level” (p. 302). Our study reflects this reality that including more meso- and macro-level factors represents a proposed avenue for future individual-focused CSR research. Thirdly, by integrating signaling theory with attribution theory, our study depicts a more complete picture regarding how different CSR perceptions impact applicants’ evaluations towards EMNEs and DMNEs (Gond et al., 2017). Fourthly, our study contributes to the advancement of knowledge about individual-level outcomes of CSR by examining the influences of individual traits, particularly skepticism (Aguinis & Glavas, 2012). Finally, by using the behavioral experiment method and leveraging the power of digital technology, we offer the CSR community a new measure to assess applicants’ awareness of companies’ CSR activities. While CSR can only exert an impact if applicants are aware of it, little attention has been given to this issue.

## Practical Implications

Our findings provide several managerial implications. We found that job-pursuit intention was significantly increased when applicants were aware of EMNEs’ good CSR. Therefore, we advise managers of EMNEs to get more engaged in good CSR with tangible and measurable impacts to overcome the less favorable country-of-origin image in hiring local talent in more developed countries. No significant difference in job-pursuit intention between mediocre CSR aware group and CSR unaware group clearly shows that it would not be beneficial for EMNEs if they only use ‘talk’ strategy without showing concrete indicators of impacts, especially when applicants tend to be more skeptical about EMNEs’ CSR. Moreover, capturing the widespread CSR claims, stakeholders are becoming more skeptical. There are also an increasing number of CSR rating agencies, watchdog groups, indices, websites dedicating to identify greenwashing (e.g., by Corpwatch, Greenpeace) as well as consumer education schemes in the form of magazines and blogs that help poeple monitor firms’ CSR performances (Pope & Wraas, 2016). If EMNEs try to take a short cut by communicating about their CSR without real actions or impacts, they will most likely not succeed.

Our findings are also important for foreign DMNEs. Particularly, the perception of country-of-origin image is crucial to DMNEs. Thus, DMNEs should emphasize their positive country-of-origin image in international recruitment. However, they should not underestimate their rival EMNEs in the war for talent. Although country-of-origin image signaling is proved to be more favorable for DMNEs and applicants are more attracted to DMNEs than to EMNEs, the gap is smaller than expected. According to several authors, it is just a matter of time when EMNEs catch up with DMNEs (Awate et al., 2015; Held & Bader, 2018). Besides, it should be noted that our findings do not imply that DMNEs should not engage in CSR activities. In contrast, we recommend that DMNEs should be diligent when develop their CSR strategy. Due to the complexity of strategic CSR and corporate brand, applicants may have higher expectations for DMNEs, so DMNEs need to address the highest set of global expectations. A violation of expectations could be seriously detrimental to DMNEs’ attractiveness as a potential employer.

Both EMNEs and DMNEs should be mindful of applicants’ skepticism as potential applicants may perceive the genuinity of CSR with skepticism, which will result in lower job-pursuit intention. Thus, companies should try to find a way to communicate their CSR activities that minimizes applicants’ skepticism. Moreover, companies cannot yield any good returns to CSR if applicants are completely unaware of their CSR activities. Thus, both EMNEs and DMNEs should find ways to improve the low CSR awareness among applicants. One way to do that is to put some hightlights of CSR activities at a prominent position on the front page of corporate websites and recruitment messages.

## Limitations and Future Research

The study has certain limitations that should be noted. First, we relied on single-source data at one point in time to test our hypotheses, which potentially raises concerns about a common method variance. Although the results suggested that CMV did not pose a major threat to the robustness of the study’s findings, future research may better counter the CMV issue by using multi-sourced methods of data collection and longitudinal data (Podsakoff et al., 2012). Second, while our study investigates variables on three levels macro-level, firm-level and micro-level -, all of them were measured as individual perceptions. In future studies, different measurements for the three levels could be used to develop a real multi-level study. By collecting real-world data, future research could also give insights further into the impacts of the home country reputations, such as ratings on the Corruption Perception Index or Reputation Institute’s CountryRep™ or Anholt-GfK Roper Nation Brands Index, on the reputations and the attractiveness of MNEs. Third, the MNEs’ webpages contained more information in the good CSR condition than in the other two conditions. Thus, there might be possible that the effects of CSR may have instead been the result of simply being exposed to more information. We ruled out this alternative explanation by measuring precisely respondents’ perceptions of the MNE’s CSR. Future research might investigate the mechanisms through which CSR enhances job-pursuit intention to provide further support for our present findings. Fourth, although we confirmed our expected moderating effect of CSR perception, we did not expect the situation which combines DMNE’s high CSR perception and low sincere motive attribution, resulting in lower job-pursuit intention. Given the complexity of strategic CSR for IB, further research should reflect on applicants’ expectations. Besides, future research may take a qualitative approach to pursue a deeper understanding of how applicants perceive corporate brands in emerging and developed countries. Fifth, this study used scenarios with five CSR dimensions, but it did not test each dimension of CSR separately. As shown in the preliminary check, these five dimensions are not equally important to applicants. Thus, it would be fruitful to replicate the study using the sub-dimensions of CSR to investigate different influences of each dimension on job-pursuit intention. Sixth, this study focused on the effects of good, mediocre, and poor CSR, but it did not investigate the relative importance of CSR compared to instrumental attributes such as salary. Future research may wish to take a step further by investigating a more complete set of job attributes. In addition, we did not take industry differences into account. The perceived attractiveness of EMNEs is very likely to differ between various industries (Holtbrügge & Kreppel, 2015). For instance, Indian IT firms have a better reputation in Western markets than carmakers of this country. Thus, future work could discuss the impacts of industry types and introduce the concept of industry image, next to country of origin. Finally, we collected cross-national data in three countries, yet we did not compare between those three. Follow-up research is needed to make cross-cultural comparisons with more sophisticated comparative methodologies. Furthermore, while the study shows that CSR influences the size of an EMNE’s applicant pool and hence selection system utility, a more practically relevant question for future research is whether CSR influences applicant pool characteristics, or, more precisely, whether CSR helps EMNEs attract not only more applicants, but also better applicants. Our sample only consists of young and highly educated individuals. It is recommended that further research should include a broader sample and measure intellectual abilities to examine whether strong applicants are more willing to look for employers’ CSR and use CSR to distinguish among potential employers.

## References

[CR1] Aguinis H (2004). Regression analysis for categorical moderators.

[CR2] Aguinis H, Glavas A (2012). What we know and don't know about corporate social responsibility: A review and research agenda. Journal of Management.

[CR3] Aiman-Smith L, Bauer TN, Cable DM (2001). Are you attracted? Do you intend to pursue? A recruiting policy-capturing study. Journal of Business and Psychology.

[CR4] Alnıaçık E, Alnıaçık Ü, Erat S, Akçin K (2014). Attracting talented employees to the company: Do we need different employer branding strategies in different cultures?. Procedia-Social and Behavioral Sciences.

[CR5] Ashforth BE, Gibbs BW (1990). The double-edge of organizational legitimation. Organization Science.

[CR6] Ashforth BE, Mael F (1989). Social identity theory and the organization. Academy of Management Review.

[CR7] Awate S, Larsen MM, Mudambi R (2015). Accessing vs sourcing knowledge: A comparative study of R&D internationalization between emerging and advanced economy firms. Journal of International Business Studies.

[CR8] Bachmann P, Ingenhoff D (2016). Legitimacy through CSR disclosures? The advantage outweighs the disadvantages. Public Relations Review.

[CR9] Backhaus KB, Stone BA, Heiner K (2002). Exploring the relationship between corporate social performance and employer attractiveness. Business and Society.

[CR10] Baum M, Kabst R (2013). How to attract applicants in the Atlantic versus the Asia-Pacific region? A cross-national analysis on China, India, Germany, and Hungary. Journal of World Business.

[CR11] Beach LR (1993). Image theory - an alternative to normative decision-theory. Advances in Consumer Research.

[CR12] Behrend TS, Baker BA, Thompson LF (2009). Effects of pro-environmental recruiting messages: The role of organizational reputation. Journal of Business and Psychology.

[CR13] Bhattacharya CB, Sen S, Korschun D (2008). Using corporate social responsibility to win the war for talent. MIT Sloan Management Review.

[CR14] Buhmann A, Ingenhoff D (2015). The 4D model of the country image: An integrative approach from the perspective of communication management. International Communication Gazette.

[CR15] Cable DM, Turban DB (2001). Establishing the dimensions, sources, and value of job seekers' employer knowledge during recruitment. Research in Personnel and Human Resources Management.

[CR16] Cadotte ER, Woodruff RB, Jenkins RL (1987). Expectations and norms in models of consumer satisfaction. Journal of Marketing Research.

[CR17] Carroll AB, Lipartito KJ, Post JE, Werhane PH (2012). Corporate responsibility: The American experience.

[CR18] Clark S, Hoque S (2012). Debating a post-American world.

[CR19] Cobanoglu C, Cavusoglu M, Turktarhan G (2021). A beginner’s guide and best practices for using crowdsourcing platforms for survey research: The Case of Amazon Mechanical Turk (MTurk). Journal of Global Business Insights.

[CR20] Contractor FJ (2013). Punching above their weight. International Journal of Emerging Markets.

[CR21] Crifo P, Diaye MA, Pekovic S (2016). CSR related management practices and firm performance: An empirical analysis of the quantity-quality trade-off on French data. International Journal of Production Economics.

[CR22] Dawkins J (2005). Corporate responsibility: The communication challenge. Journal of Communication Management.

[CR23] Deloitte. (2019). Global Millennial Survey. https://www2.deloitte.com/global/en/insights/topics/talent/deloitte-millennial-survey-2019.html

[CR24] Deloitte. (2020). Global Millennial Survey. https://www2.deloitte.com/content/dam/Deloitte/global/Documents/About-Deloitte/deloitte-2020-millennial-survey.pdf

[CR25] Devang A, Kruse C, Parker A, Siren P (2017). The next wave of business models in Asia. MIT Sloan Management Review.

[CR26] Donia MBL, Ronen S, Sirsly CAT, Bonaccio S (2019). CSR by any other name? The differential impact of substantive and symbolic CSR attributions on employee outcomes. Journal of Business Ethics.

[CR27] Dorrough AR, Glockner A (2016). Multinational investigation of cross-societal cooperation. Proceedings of the National Academy of Sciences of the United States of America.

[CR28] Du SL, Bhattacharya CB, Sen S (2007). Reaping relational rewards from corporate social responsibility: The role of competitive positioning. International Journal of Research in Marketing.

[CR29] Du SL, Bhattacharya CB, Sen S (2010). Maximizing business returns to corporate social responsibility (CSR): The role of CSR communication. International Journal of Management Reviews.

[CR30] Elving WJ (2013). Scepticism and corporate social responsibility communications: The influence of fit and reputation. Journal of Marketing Communications.

[CR31] EMF. (2013). Towards the Circular Economy Vol. 1: an economic and business rationale for an accelerated transition. Ellen McArthur Foundation. https://www.ellenmacarthurfoundation.org/assets/downloads/publications/Ellen‐MacArthur‐Foundation‐Towards‐the‐Circular‐Economy‐vol.1.pdf

[CR32] Evans WR, Davis WD (2011). An examination of perceived corporate citizenship, job applicant attraction, and CSR work role definition. Business and Society.

[CR33] Forbes. (2022). The Global 2000. Retrieved August 7, 2022 from https://www.forbes.com/lists/global2000/?sh=503c03c35ac0

[CR34] Forehand MR, Grier S (2003). When is honesty the best policy? The effect of stated company intent on consumer skepticism. Journal of Consumer Psychology.

[CR35] Froese FJ, Vo A, Garrett TC (2010). Organizational attractiveness of foreign-based companies: A country of origin perspective. International Journal of Selection and Assessment.

[CR36] FTSE. (2019). Equity Country Classification. https://www.ftserussell.com/equity-country-classification. Retrieved 07 Oct 2019.

[CR37] Fuller CM, Simmering MJ, Atinc G, Atinc Y, Babin BJ (2016). Common methods variance detection in business research. Journal of Business Research.

[CR38] Gond JP, El Akremi A, Swaen V, Babu N (2017). The psychological microfoundations of corporate social responsibility: A person-centric systematic review. Journal of Organizational Behavior.

[CR39] González-Benito J, González-Benito Ó (2006). A review of determinant factors of environmental proactivity. Business Strategy and the Environment.

[CR40] Goodman JK, Cryder CE, Cheema A (2013). Data collection in a flat world: The strengths and weaknesses of mechanical Turk samples. Journal of Behavioral Decision Making.

[CR41] Goodman JK, Paolacci G (2017). Crowdsourcing consumer research. Journal of Consumer Research.

[CR42] Great-Place-to-Work. (2018). The world’s best workplaces 2018. https://www.en.greatplacetowork.ch/best-worklaces/the-worlds-best-workplaces/2018/

[CR43] Greening DW, Turban DB (2000). Corporate social performance as a competitive advantage in attracting a quality workforce. Business and Society.

[CR44] Gully SM, Phillips JM, Castellano WG, Han K, Kim A (2013). A mediated moderation model of recruiting socially and environmentally responsible job applicants. Personnel Psychology.

[CR45] Han CM (1989). Country image - halo or summary construct. Journal of Marketing Research.

[CR46] Han CM (2015). Consumer expectations of corporate social responsibility of foreign multinationals in Korea. Emerging Markets Finance and Trade.

[CR47] Held K, Bader B (2018). The influence of images on organizational attractiveness: Comparing Chinese, Russian and US companies in Germany. International Journal of Human Resource Management.

[CR48] Held K, Berg N (2015). Liability of emergingness of emerging market multinationals in developed markets: A conceptual approach. Experiences of emerging economy firms.

[CR49] Hennart JF (2012). Emerging market multinationals and the theory of the multinational enterprise. Global Strategy Journal.

[CR50] Highhouse S, Lievens F, Sinar EF (2003). Measuring attraction to organizations. Educational and Psychological Measurement.

[CR51] Holtbrügge D, Kreppel H (2015). Employer attractiveness of chinese, indian and russian firms in Germany: Signaling effects of HR practices. Corporate Reputation Review.

[CR52] Hong G, Kim E (2017). Overcoming country-of-origin image constraints on hiring: The moderating role of CSR. Asian Business and Management.

[CR53] Janssens W, De Pelsmacker P, Wijnen K, Van Kenhove P (2008). Marketing research with SPSS.

[CR54] Jensen M, Kim H, Kim BK (2012). Meeting expectations: A role-theoretic perspective on reputation. The Oxford handbook of corporate reputation.

[CR55] Jones DA, Newman A, Shao R, Cooke FL (2019). Advances in employee-focused micro-level research on corporate social responsibility: situating new contributions within the current state of the literature. Journal of Business Ethics.

[CR56] Jones DA, Willness CR, Madey S (2014). Why are job seekers attracted by corporate social performance? Experimental and field tests of three signal-based mechanisms. Academy of Management Journal.

[CR57] Joo YR, Moon HK, Choi BK (2016). A moderated mediation model of CSR and organizational attractiveness among job applicants Roles of perceived overall justice and attributed motives. Management Decision.

[CR58] Kearney. (2019). The 2019 Kearney Foreign Direct Investment Confidence Index. https://www.kearney.com/foreign-direct-investment-confidence-index/2019-full-report. Retrieved 22 July 2019.

[CR59] Kim S, Lee YJ (2012). The complex attribution process of CSR motives. Public Relations Review.

[CR60] Kim SY, Park H (2011). Corporate social responsibility as an organizational attractiveness for prospective public relations practitioners. Journal of Business Ethics.

[CR61] KPMG. (2017). KPMG international survey of corporate responsibility reporting. https://home.kpmg/content/dam/kpmg/campaigns/csr/pdf/CSR_Reporting_2017.pdf. Retrieved 22 July 2019.

[CR62] Lagarde, C. (2016). The Role of Emerging Markets in a New Global Partnership for Growth https://www.imf.org/en/News/Articles/2015/09/28/04/53/sp020416#:~:text=As%20a%20group%2C%20emerging%20and,half%20only%20a%20decade%20ago.&text=They%20contributed%20more%20than%2080,jobs%20in%20advanced%20economies%2C%20too.

[CR63] Law KS, Song LJ, Wong CS, Chen DH (2009). The antecedents and consequences of successful localization. Journal of International Business Studies.

[CR64] Lowry PB, Gaskin JE, Twyman NW, Hammer B, Roberts TL (2013). Taking "Fun and Games" seriously: proposing the hedonic-motivation system adoption model (HMSAM). Journal of the Association for Information Systems.

[CR65] Luo YD, Tung RL (2018). A general theory of springboard MNEs. Journal of International Business Studies.

[CR66] ManpowerGroup. (2022). The talent shortage survey. https://go.manpowergroup.com/talent-shortage

[CR67] Maon F, Vanhamme J, De Roeck K, Lindgreen A, Swaen V (2019). The dark side of stakeholder reactions to corporate social responsibility: tensions and micro-level undesirable outcomes. International Journal of Management Reviews.

[CR68] Mazboudi M, Sidani YM, Al Ariss A (2020). Harmonization of firm CSR policies across national contexts: Evidence from Brazil & Sweden. International Business Review.

[CR69] Mellis AM, Bickel WK (2020). Mechanical Turk data collection in addiction research: Utility, concerns and best practices. Addiction.

[CR70] Meyer KE, Xin KR (2018). Managing talent in emerging economy multinationals: Integrating strategic management and human resource management. International Journal of Human Resource Management.

[CR71] Mitra R, Green RJ, Dutta MJ (2013). Corporate reputation in emerging markets: A culture-centered review and critique. Handbook of communication and corporate reputation.

[CR72] Mohr LA, Webb DJ, Harris KE (2001). Do consumers expect companies to be socially responsible? The impact of corporate social responsibility on buying behavior. Journal of Consumer Affairs.

[CR73] Montgomery DB, Ramus CA (2011). Calibrating MBA job preferences for the 21st century. Academy of Management Learning and Education.

[CR74] Mullen MR (1995). Diagnosing measurement equivalence in cross-national research. Journal of International Business Studies.

[CR75] Podsakoff PM, MacKenzie SB, Podsakoff NP (2012). Sources of method bias in social science research and recommendations on how to control it. Annual Review of Psychology.

[CR76] Polonsky M, Jevons C (2009). Global branding and strategic CSR: An overview of three types of complexity. International Marketing Review.

[CR77] Pope S, Wraas A (2016). CSR-washing is rare: A conceptual framework, literature review, and critique. Journal of Business Ethics.

[CR78] Rupp DE, Shao RD, Skarlicki DP, Paddock EL, Kim TY, Nadisic T (2018). Corporate social responsibility and employee engagement: The moderating role of CSR-specific relative autonomy and individualism. Journal of Organizational Behavior.

[CR79] Rupp DE, Shao R, Thornton MA, Skarlicki DP (2013). Applicants' and employees' reactions to corporate social responsibility: The moderating effects of first-party justice perceptions and moral identity. Personnel Psychology.

[CR80] Rynes, S. L. (1989). Recruitment, job choice, and post-hire consequences: A call for new research directions.***

[CR81] Schwab, K. (2019). The Global Competitiveness Report 2019. World Economic Forum. http://www3.weforum.org/docs/WEF_TheGlobalCompetitivenessReport2019.pdf

[CR82] Shirodkar V, Shete N (2022). The impact of domestic CSR on the internationalisation of emerging-market multinational enterprises: Evidence from India. Management International Review.

[CR83] Simcox T, Fiez JA (2014). Collecting response times using Amazon mechanical turk and adobe flash. Behavior Research Methods.

[CR84] Spence M (1973). Job market signaling. Quarterly Journal of Economics.

[CR85] Tajfel H (1974). Social identity and intergroup behaviour. Social Science Information Sur Les Sciences Sociales.

[CR86] Tanaka T, Camerer CF (2016). Trait perceptions influence economic out-group bias: Lab and field evidence from Vietnam. Experimental Economics.

[CR87] UNCTAD. (2019). World Investment Report. 2019. https://unctad.org/en/pages/PublicationWebflyer.aspx?publicationid=2460

[CR88] UNDP. (2019). Human Development Report 2019. http://hdr.undp.org/sites/default/files/hdr2019.pdf. Retrieved 08 Jan 2020.

[CR89] Vaara E, Tienari J (2008). A discursive perspective on legitimation strategies in multinational corporations. Academy of Management Review.

[CR90] Van de Vliert E (2011). Climato-economic origins of variation in ingroup favoritism. Journal of Cross-Cultural Psychology.

[CR91] Vandenberg RJ, Lance CE (2000). A review and synthesis of the measurement invariance literature: Suggestions, practices, and recommendations for organizational research. Organizational Research Methods.

[CR92] Vidaver-Cohen D, Gomez C, Colwell SR (2015). Country-of-origin effects and corporate reputation in multinational firms: Exploratory research in Latin America. Corporate Reputation Review.

[CR93] Yoon Y, Gurhan-Canli Z, Schwarz N (2006). The effect of corporate social responsibility (CSR) activities on companies with bad reputations. Journal of Consumer Psychology.

[CR94] Zellmer-Bruhn M, Caligiuri P, Thomas DC (2016). From the editors: Experimental designs in international business research.

[CR97] Zhang L, Gowan MA (2012). Corporate social responsibility, applicants' individual traits, and organizational attraction: A person-organization fit perspective. Journal of Business and Psychology.

[CR95] Zhang JH, He XM, Zhou CH, van Gorp D (2019). Antecedents of corporate image: The case of Chinese multinational enterprises in the Netherlands. Journal of Business Research.

[CR96] Zhang JH, Zhou CH, van Gorp DM, van Witteloostuijn A (2020). Willingness to work for multinational enterprises from emerging countries: The case of Chinese multinational enterprises in the Netherlands. International Business Review.

[CR98] Zhang Q, Cao M, Zhang FF, Liu J, Li X (2020). Effects of corporate social responsibility on customer satisfaction and organizational attractiveness: A signaling perspective. Business Ethics-A European Review.

